# Six-year clinical course of depressive and anxiety disorders: analysis by age

**DOI:** 10.1136/bmjment-2026-302895

**Published:** 2026-10-06

**Authors:** Federico Triolo, Laura K M Han, Hans W Jeuring, Erik J Giltay, Brenda W J H Penninx

**Affiliations:** 1 Aging Research Center, Department of Neurobiology, Care Sciences and Society, Karolinska Institutet, Stockholm, Sweden; 2 Department of Psychiatry, Amsterdam Univerisity Medical Center, Vrije Universiteit, Amsterdam, The Netherlands; 3 Amsterdam Neuroscience, Amsterdam, The Netherlands; 4 Department of Psychiatry, University Medical Center Groningen, Groningen, The Netherlands; 5 Department of Psychiatry, Leiden University Medical Center, Leiden, The Netherlands

**Keywords:** Depressive Disorder, Anxiety Disorders

## Abstract

**Background:**

Evidence on the association between age and the long-term course of affective disorders remains mixed.

**Objective:**

This study examined the 6-year symptom and diagnostic trajectories of depression and anxiety in relation to age, and assessed whether social, lifestyle, somatic and clinical factors explained observed age-related differences.

**Methods:**

A cohort of 1701 participants aged 18–65 years with a current depressive and/or anxiety disorder from the Netherlands Study of Depression and Anxiety were assessed biennially over 6 years. Clinical diagnoses were ascertained using the Composite International Diagnostic Interview, while symptom severities were measured with the Inventory of Depressive Symptomatology, Beck Anxiety Inventory, Fear Questionnaire and Penn State Worry Questionnaire. Linear mixed-effects models examined age differences in symptom changes over time; multinomial logistic regression examined age differences in diagnostic course. Models were sequentially adjusted for social, lifestyle, somatic and clinical factors to explain observed age-related differences.

**Findings:**

Over the 6-year follow-up, older age was associated with slower improvement in depressive (b_per 10 years_=0.191 (0.103 to 0.279)) and anxiety symptoms (b_per 10 years_=0.119 (0.048 to 0.191)), but not for fear or worry. Older age was associated with a higher likelihood of a chronic course versus early remission (RRR_per 10 years_=1.19 (1.05 to 1.36)), primarily driven by depression chronicity. Age-related differences in symptom trajectories persisted across all adjusted models, while associations with depression diagnostic chronicity were attenuated after adjustment for social and lifestyle factors.

**Conclusions:**

Higher chronological age is an independent risk factor for a less favourable long-term course of affective disorders, particularly depression, characterised by slower symptom improvement and increased chronicity.

**Clinical implications:**

Age-tailored depression management, including closer follow-up and timely treatment optimisation, may help improve clinical course in later life.

**WHAT IS ALREADY KNOWN ON THIS TOPIC**

- Evidence on the association between age and the course of affective disorders is mixed.
- Most studies have focused on depression and short-term outcomes, leaving uncertainty about the impact of age on the long-term course of depression and anxiety and the factors underlying age-related differences.

**WHAT THIS STUDY ADDS**

- In a 6-year prospective cohort of adults with depressive and/or anxiety disorders, older age was associated with slower improvement in depressive and anxiety symptoms and a higher likelihood of a chronic course, particularly for depression.
- These associations persisted after accounting for social, lifestyle, somatic and clinical factors, suggesting that chronological age is an independent risk factor for poorer long-term outcomes.

**HOW THIS STUDY MIGHT AFFECT RESEARCH, PRACTICE OR POLICY**

- These findings support age-tailored management of depression across adulthood, particularly in older individuals, and foster research on the mechanisms underlying age-related differences in the course of affective disorders.

## Background

The long-term course of depressive and anxiety disorders is often unfavourable.^1^ Recurrence, chronicity and incomplete recovery are common, and their cumulative consequences, including somatic illness, functional impairment and premature mortality, make prognosis a central public health concern.^2 3^

Chronological age reflects biological, psychological, social changes across the life course that are likely to influence the course of affective disorders.^2^ Evidence on whether older age predicts a less favourable course is, however, mixed: some studies report no significant age-related differences in outcome,^4–6^ while others suggest that ageing may even confer advantages in terms of disease course.^7–10^ Where worse outcomes have been observed, older age has been associated with higher chronicity, greater symptom burden and longer time to remission over 2-year follow-up, both in a cohort partly overlapping with the present sample^11^ and in independent community and primary care samples of depression.^12 13^ Discrepancies between studies likely reflect differences in design, follow-up duration, sample composition (clinical vs population-based) and outcome definitions, as well as variation in the extent to which factors that covary with ageing are accounted for. For example, several characteristics become increasingly prevalent with age, including poorer somatic health, reduced social connectedness, greater loneliness and lower levels of physical activity, and are themselves associated with less favourable outcomes in affective disorder. It therefore remains unclear whether chronological age independently shapes the long-term course of affective disorders beyond social, somatic and lifestyle factors.

Another knowledge gap in the existing literature is its predominant focus on depression, with anxiety disorders receiving less attention.^6 14–16^ This is particularly notable given the high comorbidity between depression and anxiety, the frequent transitions between these conditions over time, and evidence that comorbidity is associated with worse outcomes than either disorder alone. Whether age influences the course of anxiety disorders differently than depression, and whether anxiety-specific symptom dimensions such as somatic anxiety, fear and pathological worry show distinct age-related trajectories, remain unknown. Examining both conditions jointly and separately, and using both diagnostic and symptom outcome measures, is essential for a comprehensive account of how ageing influences the prognosis of affective disorders.

The present study addresses these gaps using data from the Netherlands Study of Depression and Anxiety (NESDA), a large naturalistic cohort with detailed clinical assessments over time and a broad age range at baseline (18–65 years). We examined associations between chronological age and 6-year course of affective disorders across two complementary outcome domains: dimensional symptom trajectories and categorical diagnostic course. Further, we assessed whether any age-related difference was attributable to factors that vary with ageing, including somatic health, social characteristics, lifestyle and clinical factors. We hypothesised that older age would be associated with a less favourable long-term course of affective disorders, which could be partially explained by age-related somatic and social characteristics.

## Methods

Data were drawn from NESDA, an ongoing longitudinal cohort study examining the naturalistic course of depressive and anxiety disorders (for the full study protocol, see previous work^17^). At baseline (2004–2007), a total of 2981 participants aged between 18 and 65 years were recruited from the community (19%), primary care (54%) and specialised mental healthcare (27%), of whom 57.1% had an affective disorder diagnosis. Face-to-face follow-up assessments were conducted at 2 years (87.1%, n=2596), 4 years (80.6%, n=2402), 6 years (75.5%, n=2256), with the 6-year follow-up being completed in 2013. For the present study, we included 1701 participants who met diagnostic criteria according to the Diagnostic and Statistical Manual of Mental Disorders - IV edition (DSM-IV) for a depressive disorder (ie, major depressive disorder (MDD) or dysthymia), and/or an anxiety disorder (panic disorder, social phobia, generalised anxiety disorder and/or agoraphobia) in the 6 months prior to baseline and had follow-up information available during the 6-year observation period. Baseline characteristics of participants retained during the entire follow-up and those who dropped out are presented in online supplemental Table S1.

### Measures

#### Symptom course (dimensional)

Depressive and anxiety symptom severity was assessed at baseline and all follow-up waves to examine dimensional trajectories of affective symptoms. Depressive symptom severity was measured using the Inventory of Depressive Symptomatology – Self-Report (IDS). Anxiety symptom severity was assessed using the Beck Anxiety Inventory (BAI), the Fear Questionnaire (FQ) and the Penn State Worry Questionnaire (PSWQ), capturing complementary domains of somatic anxiety, fear and pathological worry.

#### Diagnostic course (categorical)

Diagnostic course was operationalised following a previously developed approach.^18^ Briefly, DSM-IV diagnoses of depressive and anxiety disorders were ascertained using the Composite International Diagnostic Interview (CIDI) V.2.1, administered in person by trained research personnel at baseline and at each follow-up wave (2, 4 and 6 years). To assess whether disorders were continuously present during follow-up intervals, symptom continuity was evaluated using the Life Chart Interview (LCI). The LCI anchors retrospective symptom reporting to age-linked and calendar-linked personal landmarks to enhance recall accuracy. Continuous symptomatology was defined as the presence of symptoms during at least 85% of the months between two assessments (ie, no more than two symptom-free months).

At baseline, participants were categorised into three mutually exclusive groups: (1) depression only (MDD, dysthymic disorder, or both), (2) anxiety only (panic disorder, social phobia, generalised anxiety disorder, agoraphobia, or any combination thereof), and (3) comorbid depression and anxiety. Categorical diagnostic trajectories were defined at 2-year, 4-year and 6-year follow-up based on the presence of any CIDI diagnosis at each assessment, supplemented by continuity of symptoms from the LCI collected between assessments. Participants were assigned to one of four mutually exclusive disease course categories: *early remission* (no diagnosis at any follow-up), *late remission* (no diagnosis at the most recent follow-up, but with at least one diagnosis at an earlier follow-up assessment), *recurrent* (diagnosis present at the most recent follow-up, but not continuously across all follow-up assessments) and *chronic* (diagnosis present at all follow-ups, with continuous LCI symptoms throughout). This classification was first applied to any depressive or anxiety diagnosis across the sample with diagnostic data in at least one follow-up wave after baseline (n=1419). To distinguish between disorder-specific trajectories, the same classification was subsequently applied within the subsample of participants with a depressive disorder at baseline (n=946) and within those with an anxiety disorder at baseline (n=1075), using only the index disorder to determine course category. For all three outcomes (affective disorder course, depression course and anxiety course), each participant’s outcome was based on the latest post-baseline assessment for which CIDI and LCI data were available within the 6-year follow-up window rather than requiring data at all follow-up waves.

#### Explanatory factors

To examine whether age-related differences in the course of affective disorders could be explained by social, lifestyle, somatic health and clinical characteristics, we included baseline covariates drawn from prior literature on the prognosis of affective disorders.^2 3^ Social factors comprised partner status (married or cohabiting vs not), loneliness (11-item Loneliness and Affiliation Scale; range 0–11, with higher scores indicating greater loneliness) and social network size (number of regular and important contacts), capturing both structural and subjective aspects of social life. Lifestyle factors included physical activity level (high level (vigorous-intensity activity on ≥3 days achieving ≥1500 MET-min/week, or a combination of walking, moderate and vigorous activity on ≥7 days achieving ≥3000 MET-min/week) vs moderate or low) assessed through the International Physical Activity Questionnaire (IPAQ), smoking (pack-year) and alcohol use (units/week). Somatic health indicators included body mass index (BMI; kg/m²), number of self-reported chronic somatic diseases (ie, lung disease, heart disease, diabetes, stroke, osteoarthritis, cancer, ulcers, intestinal disease, liver disease, epilepsy and thyroid disease) and pain severity measured with the Chronic Pain Grade (CPG; range 0–4, with higher scores indicating more severe and disabling pain). Clinical characteristics included symptom severity (IDS, BAI, FQ and PSWQ), baseline diagnoses and treatment-related variables: use of antidepressants, benzodiazepine and/or other psychotropic medications and psychotherapy (all yes/no).

### Statistical analyses

In all primary analyses, age at baseline was modelled as a continuous variable. For interpretability, effect estimates are presented per 10-year increase in age. To further illustrate age-related differences across the adult lifespan, descriptive and graphical results were additionally presented for four age categories: 18–29, 30–39, 40–49 and 50–65 years.

Baseline characteristics were tabulated across age groups and associated with continuous age in linear regressions, with each factor as the independent variable and age as the dependent variable. Factors significantly associated with age were subsequently entered into sequential models to assess the extent to which they accounted for the association between age and disease course.

Longitudinal associations between age at baseline and change in symptomatology (IDS, BAI, FQ and PSWQ) were examined using linear mixed-effects models with repeated observations nested within individuals. All models included fixed effects for age at baseline, follow-up time and their interaction, such that the interaction term reflects the difference in rate of symptom change over follow-up per 10-year increase in baseline age. To account for within-person correlation and inter-individual heterogeneity in symptom trajectories, random intercepts and slopes for follow-up time were specified at the participant level; model fit indices (ie, log-likelihoods) indicated that inclusion of random slopes significantly improved the model fit (online supplemental Table S2).

Multinomial logistic regression was used to examine associations between age and diagnostic course (early remission (reference), late remission, recurrent, chronic). Relative risk ratios (RRRs) with 95% CIs were estimated, and predicted probabilities with bootstrapped CIs were derived and plotted across the age range (18–65 years). Analyses were conducted for overall affective disorder course and separately for depressive and anxiety disorders.

To assess whether age-related associations were explained by factors that tend to vary with age, adjustment was examined separately by covariate domain, which allowed to isolate the contribution of each domain. For the symptom change analysis, a series of mixed-effects models were fitted additionally adjusting for the main effect of each covariate and its interaction with time, across four domains: (1) somatic health factors (number of chronic diseases, pain and BMI), (2) lifestyle factors (physical activity, alcohol use and smoking), (3) social factors (partner status, loneliness and social network size) and (4) clinical factors (use of antidepressants, benzodiazepine, other psychotropic medications and psychotherapy). A multi-adjusted model including all covariates associated with symptom change over time was also fitted, again including both their main effects and interactions with time. All models were adjusted for sex and education. For the diagnostic course analysis, four separate multinomial regression models were fitted, each additionally adjusting for the main effects of one covariate domain (clinical, somatic, lifestyle and social factors, respectively). All analyses were conducted using complete-case data (online supplemental Table S3 reports data missingness).

All statistical tests were two-sided, with p<0.05 considered statistically significant. Statistical analyses were carried out in R V.4.3.2.

## Results

Baseline characteristics across age categories and linear associations with continuous age are presented in table 1. The mean age of the sample was 41.3 years (SD=12.4), with 67% female participants.

**Table 1:** Baseline characteristics across age categories and linear associations with continuous age

	Total population n=1701	18–29 n=373	30–39 n=375	40–49 n=443	50–65 n=510	Age continuous; b (95% CI)
Sociodemographics						
Age (years)	41.3 (12.4)	24.2 (3.2)	34.5 (2.9)	44.4 (2.7)	56.0 (4.1)	–
Sex (female)	1140 (67%)	286 (77%)	266 (71%)	272 (61%)	316 (62%)	−3.01 (−4.25 to −1.76)***
Education (years)	11.8 (3.3)	12.0 (2.8)	12.3 (3.5)	11.4 (3.3)	11.8 (3.3)	−0.25 (−0.43 to −0.07)**
Social factors						
Married or life partner (yes)	1110 (65%)	203 (54%)	255 (68%)	301 (68%)	351 (69%)	2.54 (1.31 to 3.76)***
Loneliness (range 0-11)	5.8 (3.6)	4.9 (3.7)	5.5 (3.7)	6.3 (3.4)	6.1 (3.6)	0.42 (0.24 to 0.59)***
Social network size (number of contacts)	6.0 (4.6)	7.2 (4.8)	5.8 (4.1)	5.7 (4.5)	5.7 (4.9)	−0.20 (−0.33 to −0.08)**
Lifestyle factors						
Physical activity (high)	527 (33%)	137 (39%)	122 (35%)	142 (34%)	126 (27%)	−2.31 (−3.59 to −1.02)^a^***
Smoking (pack-year)	12.6 (17.5)	3.3 (4.9)	7.3 (9.9)	15.3 (16.5)	21.0 (23.1)	0.28 (0.25 to 0.31)***
Alcohol use (units/week)	6.0 (8.3)	4.8 (7.1)	4.8 (6.7)	5.9 (8.8)	7.7 (9.5)	0.20 (0.13 to 0.27)***
Somatic health						
BMI (kg/m^2^)	25.7 (5.3)	23.5 (4.9)	25.3 (5.2)	26.3 (5.4)	27.0 (5.0)	0.56 (0.45 to 0.66)***
Number of chronic diseases	1.0 (1.1)	0.6 (0.8)	0.8 (0.9)	0.9 (1.0)	1.5 (1.3)	3.59 (3.09 to 4.10)***
Pain severity (range 0–4)	1.8 (1.1)	1.6 (0.9)	1.8 (1.1)	2.0 (1.2)	1.9 (1.1)	1.07 (0.54 to 1.61)***
Clinical factors						
Depressive symptoms (IDS)	29.3 (12.5)	27.6 (12.4)	29.0 (12.1)	30.5 (13.0)	29.5 (12.2)	0.05 (0.01 to 0.10)*
Anxiety symptoms (BAI)	17.1 (10.8)	16.8 (10.5)	16.9 (11.0)	17.2 (10.8)	17.2 (10.8)	0.02 (−0.03 to 0.08)
Fear symptoms (FQ)	32.8 (20.6)	31.3 (19.7)	33.6 (20.9)	34.2 (20.9)	32.1 (20.7)	0.01 (−0.02 to 0.04)
Worry symptoms (PSWQ)	36.7 (10.1)	37.2 (10.3)	37.7 (10.2)	36.4 (10.2)	36.1 (9.6)	−0.06 (−0.13 to 0.00)
Psychiatric diagnoses						
Only depression	396 (23%)	85 (23%)	90 (24%)	113 (26%)	108 (21%)	REF
Only anxiety	543 (32%)	123 (33%)	122 (33%)	116 (26%)	182 (36%)	0.87 (−0.73 to 2.47)
Comorbid depression and anxiety	762 (45%)	165 (44%)	163 (43%)	214 (48%)	220 (43%)	0.28 (−1.22 to 1.79)
Any antidepressant use (yes)	650 (38%)	111 (30%)	135 (36%)	200 (45%)	204 (40%)	2.22 (1.01 to 3.42)***
Any benzodiazepine use (yes)	208 (12%)	14 (3.8%)	30 (8.0%)	67 (15%)	97 (19%)	7.32 (5.56 to 9.08)***
Any other psychotropic drug use (yes)	841 (49%)	137 (37%)	163 (43%)	247 (56%)	294 (58%)	4.48 (3.32 to 5.63)***
Psychotherapy	743 (44%)	187 (50%)	177 (47%)	201 (45%)	178 (35%)	−3.20 (−4.38 to −2.03)***

Data presented as mean (SD) or n (%). ^a^Reference: moderate or low physical activity. Significance: *p<0.05; **p<0.01; ***p<0.001.

BAI, Beck Anxiety Inventory; BMI, body mass index; FQ, Fear Questionnaire; IDS, Inventory of Depressive Symptomatology – Self-Report; PSWQ, Penn State Worry Questionnaire.

Older participants had a higher somatic burden, including greater BMI, more chronic diseases and higher pain levels. Further, they presented with poorer lifestyle factors (lower physical activity, higher alcohol consumption and greater smoking) and social life (greater loneliness and fewer close social contacts). Regarding clinical characteristics, they were also more likely to use psychotropic medications, particularly antidepressants and benzodiazepines, and less likely to receive psychotherapy. Baseline depressive symptom severity was broadly similar across ages, with slightly lower values among the youngest participants, whereas anxiety-related symptom levels were comparable across age groups. The distribution of baseline diagnostic categories (depression only, anxiety only and comorbid depression–anxiety) was similar across age groups.

Associations between age and symptom change over the follow-up are presented in figure 1 as predicted trajectories, adjusted for sex and education.

**Figure 1:**
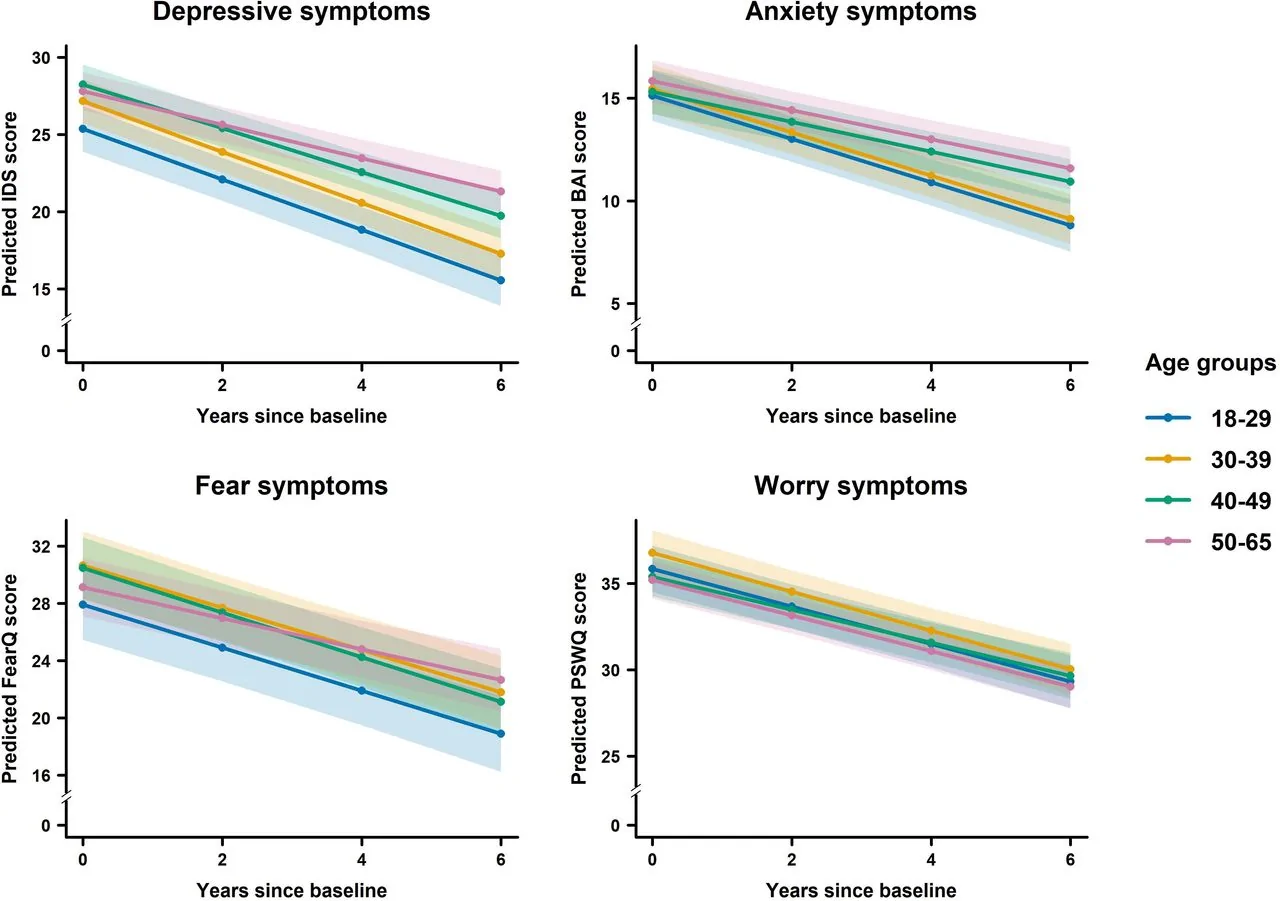
Predicted symptom trajectories by age group from linear mixed-effects models adjusted for sex and education. Compared with the 18–29 age group, the rate of depressive symptom decline over time was slower in the 50–65 age group, and the rate of anxiety symptom decline was slower in both the 40–49 and 50–65 age groups (all p<0.05). BAI, Beck Anxiety Inventory; IDS, Inventory of Depressive Symptomatology; PSWQ, Penn State Worry Questionnaire.

Across all symptom scales, symptom levels declined over time (depression: b per 10-year interval=−2.20, 95% CI −2.59 to −1.82; anxiety: b=−1.36, 95% CI −1.67 to −1.05; fear: b=−1.83, 95% CI −2.365 to −1.295; worry: b=−1.098, 95% CI −1.438 to −0.757). Higher age was associated with a higher level of depressive symptoms at baseline (b=0.66, 95% CI 0.19 to 1.11) and with slower improvement in depressive symptoms over time (b age×FU = 0.191, 95% CI 0.103 to 0.279), while anxiety symptoms were only associated with slower improvement over time (b=0.119, 95% CI 0.048 to 0.191). In contrast, age was not significantly associated with baseline or change, neither for fear (b=0.32, 95% CI −0.44 to 1.08; b age×FU=0.109, 95% CI −0.014 to 0.231) nor for worry symptoms (b=−0.31, 95% CI −0.72 to 0.10; b age×FU=0.013, 95% CI −0.065 to 0.091). All coefficients for the basic adjusted models are reported in online supplemental Table S4.

Table 2 presents coefficients for models with depressive and anxiety symptoms as outcomes, further adjusted for sets of potential explanatory factors. These associations between age and symptom change persisted across all fully adjusted models for both depressive and anxiety symptoms, with point estimates ranging from 0.191 to 0.222 for depressive symptoms and from 0.119 to 0.206 for anxiety symptoms, suggesting that none of the covariate domains explained the age associations. Regarding individual somatic factors, both pain and BMI were associated with higher baseline levels of depressive and anxiety symptoms, but also with faster symptom decline over time, whereas the number of chronic diseases was associated only with higher baseline symptom burden and not with change over time. Within the social domain, loneliness was associated with higher baseline symptom levels but faster decline over time, while having a partner was associated with faster decline in anxiety symptoms only. Among lifestyle factors, smoking was associated with higher baseline symptom levels and faster decline over time for anxiety symptoms, while physical activity was associated only with lower baseline levels of both depressive and anxiety symptoms. Among clinical factors, psychotherapy was associated with faster symptom decline, as was benzodiazepine use for anxiety specifically.

**Table 2:** Association between age and symptom change over the follow-up across adjustment models

	Depressive symptoms	Anxiety symptoms
Model 1: Basic	*n=1690*	*n=1691*
Age (per 10 years)	0.656 (0.198 to 1.113)**	0.191 (−0.197 to 0.579)
FU (years)×Age (per 10 years)	0.191 (0.103 to 0.279)***	0.119 (0.048 to 0.191)**
Model 2: Basic+Somatic factors	*n=1690*	*n=1691*
Age (per 10 years)	0.000 (−0.457 to 0.458)	−0.313 (−0.708 to 0.082)
FU (years)×Age (per 10 years)	0.222 (0.128 to 0.317)***	0.156 (0.080 to 0.232)***
Number of chronic diseases	0.826 (0.297 to 1.355)**	0.750 (0.294 to 1.207)**
FU (years)×Number of chronic diseases	0.053 (−0.059 to 0.165)	0.029 (−0.061 to 0.120)
Pain severity	3.559 (3.044 to 4.074)***	2.638 (2.194 to 3.082)***
FU (years)×Pain severity	−0.254 (−0.362 to −0.146)***	−0.189 (−0.276 to −0.102)***
BMI	0.119 (0.013 to 0.225)*	0.063 (−0.028 to 0.154)
FU (years)×BMI	−0.025 (−0.047 to −0.003)*	−0.030 (−0.048 to −0.012)***
Model 3: Basic+Lifestyle factors	*n=1565*	*n=1566*
Age (per 10 years)	0.381 (−0.131 to 0.893)	−0.192 (−0.624 to 0.240)
FU (years)×Age (per 10 years)	0.193 (0.094 to 0.292)***	0.159 (0.080 to 0.239)***
Physical activity (high vs low or moderate)	−2.949 (−4.179 to −1.720)***	−1.880 (−2.917 to −0.843)***
FU (years)×Physical activity (IPAQ)	0.124 (−0.114 to 0.363)	0.190 (−0.002 to 0.383)
Alcohol use (units/week)	−0.089 (−0.161 to −0.017)*	−0.058 (−0.119 to 0.002)
FU (years)×Alcohol use (units/week)	0.013 (−0.001 to 0.026)	0.007 (−0.004 to 0.018)
Smoking (pack-year)	0.056 (0.018 to 0.093)**	0.083 (0.051 to 0.114)***
FU (years)×Smoking (pack-year)	−0.006 (−0.013 to 0.002)	−0.010 (−0.016 to −0.004)***
Model 4: Basic+Social factors	*n=1437*	*n=1437*
Age (per 10 years)	0.267 (−0.188 to 0.723)	0.012 (−0.393 to 0.418)
FU (years)×Age (per 10 years)	0.206 (0.114 to 0.298)***	0.138 (0.063 to 0.214)***
Partner status: Partnered	0.119 (−1.080 to 1.318)	0.969 (−0.098 to 2.037)
FU (years)×Partner status: Partnered	−0.220 (−0.462 to 0.022)	−0.261 (−0.459 to −0.063)**
Loneliness	1.258 (1.088 to 1.428)***	0.661 (0.510 to 0.812)***
FU (years)×Loneliness	−0.069 (−0.104 to −0.035)***	−0.034 (−0.062 to −0.006)*
Social contacts	−0.189 (−0.317 to −0.062)**	0.018 (−0.096 to 0.131)
FU (years)×Social contacts	−0.002 (−0.028 to 0.023)	−0.015 (−0.035 to 0.006)
Model 5: Basic+Clinical factors	*n=1690*	*n=1691*
Age (per 10 years)	0.154 (−0.304 to 0.612)	−0.264 (−0.654 to 0.126)
FU (years)×Age (per 10 years)	0.208 (0.118 to 0.299)***	0.146 (0.073 to 0.220)***
Antidepressant use	0.624 (−1.254 to 2.502)	−0.316 (−1.915 to 1.282)
FU (years)×Antidepressant use	−0.244 (−0.635 to 0.146)	−0.136 (−0.451 to 0.179)
Benzodiazepine use	6.456 (4.630 to 8.283)***	6.071 (4.516 to 7.626)***
FU (years)×Benzodiazepine use	−0.242 (−0.619 to 0.134)	−0.354 (−0.658 to −0.050)*
Other psychotropic use	3.024 (1.092 to 4.955)**	2.611 (0.967 to 4.255)**
FU (years)×Other psychotropic use	−0.172 (−0.570 to 0.226)	−0.187 (−0.509 to 0.135)
Psychotherapy: Yes	1.052 (−0.069 to 2.173)	0.423 (−0.532 to 1.378)
FU (years)×Psychotherapy: Yes	−0.329 (−0.552 to −0.107)**	−0.190 (−0.370 to −0.010)*
Model 6: Multi-adjusted model	*n=1439*	*n=1428*
Age (per 10 years)	0.036 (−0.410 to 0.483)	−0.651 (−1.078 to −0.224)**
FU (years)×Age (per 10 years)	0.211 (0.117 to 0.305)***	0.206 (0.123 to 0.289)***

Cells show b (95% CI) from linear mixed models. Significance: *p<0.05; **p<0.01; ***p<0.001. FU: follow-up time in years. All models are adjusted for sex and education. In the multi-adjusted model (model 6), only main effect and their interaction with time of the selected variables are included: depressive symptom model (pain severity, BMI, loneliness, psychotherapy); anxiety symptom model (pain severity, BMI, loneliness, partner status, smoking, benzodiazepine use, psychotherapy).

BMI, body mass index; IPAQ, International Physical Activity Questionnaire.

We next examined associations between age and categorical diagnostic course. Predicted probabilities of diagnostic trajectories across age, adjusted for sex and education, are shown in figure 2 (left panel), with corresponding relative risk ratios (RRRs) in the right panels. The probability of chronicity increased gradually with age for overall affective disorders and specifically for depression, while the probability of early remission declined correspondingly. No clear age-related trends emerged for late remission or recurrent trajectories (overall affective disorder or depression), nor for any anxiety-specific probabilities. As compared with early remission, older age was associated with a higher likelihood of chronicity for overall affective disorder (RRR per 10 years=1.19, 95% CI 1.05 to 1.36). Associations were primarily driven by depression and not anxiety clinical course trajectories. As compared with early remission, older age was associated with depression chronicity (RRR=1.30, 95% CI 1.09 to 1.56) and late remission depression (RRR=1.19, 95% CI 1.03 to 1.36). On adjustment for explanatory factor domains (online supplemental Table S5), the association between age and chronicity for depression remained similar after adjustment for clinical and somatic factors, but was attenuated in models adjusted for lifestyle and social factors. No significant associations were observed for anxiety-specific course (RRRs ~1, for all disease courses, right panel of figure 2).

**Figure 2:**
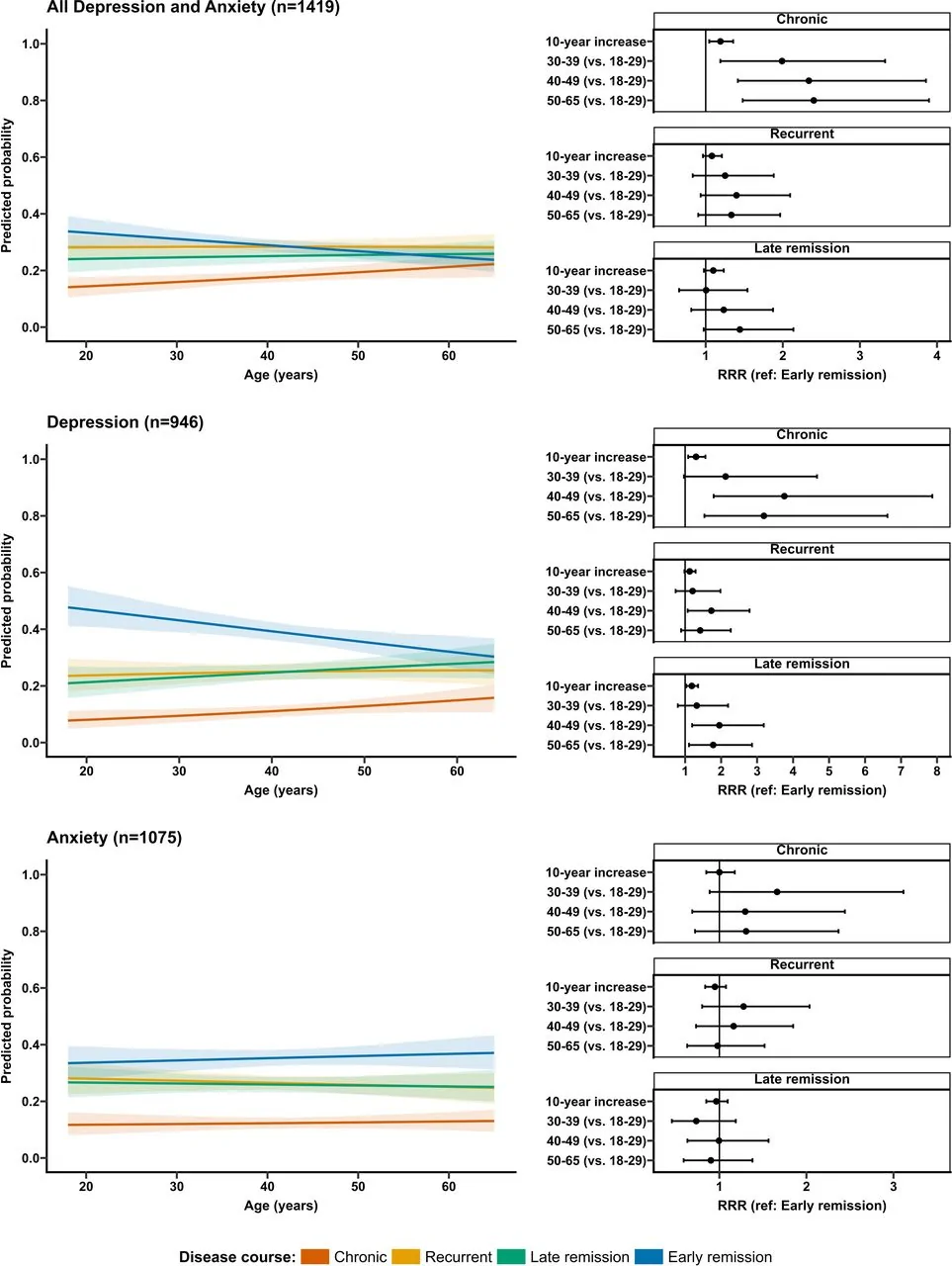
Predicted marginal probabilities of disease course by age (left panels) and corresponding relative risk ratios (RRRs) for age from multinomial regression models (right panels; reference group: early remission) adjusted for sex and education.

## Discussion

In this 6-year longitudinal study of adults with a current affective disorder, including depressive and/or anxiety disorders, higher chronological age was associated with a less favourable symptom course and increased diagnostic chronicity. Older age predicted a slower decline in depressive and anxiety symptoms over time, whereas trajectories of fear and worry symptoms did not differ by age. In addition, older individuals were more likely to follow a chronic diagnostic course rather than early remission, an association driven by chronicity in depression rather than anxiety. These patterns remained largely consistent after adjustment for a broad range of social, lifestyle, somatic and clinical characteristics, although associations with diagnostic course were attenuated after adjustment for social and lifestyle factors. Taken together, the findings suggest that the naturalistic long-term course of depression becomes less favourable with higher age, whereas age effects on the course of anxiety disorders appear more limited, with the exception of anxiety symptoms.

### Depression

At baseline, higher age was associated with greater overall depressive symptom severity. Although this differs from studies reporting greater symptom burden at younger ages, age-related differences may also concern symptom composition rather than overall severity. Previous research examining the clinical presentation of depression across the adult lifespan (ages 18–88) found relatively more affective/cognitive symptoms at younger ages and more somatic symptoms at older ages, despite no differences in overall severity.^19^ A relatively more somatic presentation of depression at older ages has also been reported elsewhere.^20^ Thus, overall symptom severity scores may not necessarily reflect age-related differences in specific symptom domains.

The finding that older age was associated with a slower decline in depressive symptoms and a greater likelihood of chronicity over 6 years extends previous NESDA findings based on shorter follow-up periods on depression^11^ and overall affective disorders,^3^ suggesting that age-related differences persist even over extended follow-up time. Evidence regarding age differences in the course of depression nonetheless remains mixed. Some studies find no significant association between age and depression outcomes, including one examining naturalistic 2-year remission among antidepressant users^4^ and a meta-analysis of RCTs in primary care that found no association between age and symptom improvement over 3–4 months.^5^ These null findings may partly reflect the shorter follow-up periods, greater baseline severity in participants of randomized controlled trials, and the structured treatment contexts involved, where symptom change is likely driven more by treatment intensity than by ageing processes. Notably, several investigations from one population-based study have reported findings in the opposite direction, with younger age associated with poorer prognosis across follow-up periods of varying length.^7–10^ These contrasting findings may partly reflect differences in recruitment setting and sample composition. Population-based samples include individuals across a wide spectrum of symptom severity, whereas clinical cohorts like NESDA are disproportionately enriched for individuals with more severe and complex clinical presentations,^17^ among whom age-related differences in prognosis may be more pronounced. Overall, our results suggest that higher age is associated with a less favourable depression course in naturalistic clinical settings. At the same time, the heterogeneity of findings across studies suggests that age-related differences in depression prognosis may be context-dependent rather than uniform across settings.

### Anxiety

With regard to anxiety disorders, we found limited evidence for age-related differences in the long-term course, in contrast to depression. Age was not associated with diagnostic chronicity, nor with trajectories of fear or pathological worry. The one exception was anxiety as measured by the BAI, which captures the more somatic arousal aspects of anxiety, with older age being associated with a slightly slower symptom decline. This pattern may reflect the closer link between somatic anxiety and physical morbidity, which increases with age and may sustain physiological arousal.^21^ Nonetheless, adjustment for somatic health factors did not modify the results, indicating that the observed age effect extends beyond physical morbidity. Overall, the absence of age effects on anxiety course is consistent with previous NESDA findings showing no association between age and a more chronic 2-year anxiety course based on life chart assessments,^14^ and with a population-based study of panic disorder in which age was unrelated to the course of panic attacks over time.^6^ In contrast, two studies reported more favourable anxiety course with older age. A 6-year population-based study found higher age to be associated with lower recurrence,^16^ while a 14-year outpatient follow-up study found faster symptom decline in panic disorder and generalised anxiety disorder, although not in social phobia,^15^ suggesting differential effects across different anxiety disorders. Overall, these results suggest that the long-term course of anxiety disorders appears largely independent of chronological age, with the possible exception of somatic anxiety symptoms.

### Age associations beyond explanatory factors

Although older participants presented with a more adverse profile across social, somatic and lifestyle domains, adjustment for these factors did not account for the association between age and symptom trajectories. This suggests that the observed age-related differences in symptom improvement were not attributable to baseline differences in risk factors that accompany ageing. Notably, several adverse factors, including pain and loneliness, were associated with higher baseline symptom levels but also with faster improvement over time. This may reflect greater room for improvement and differences in clinical attention and treatment intensity among individuals with higher baseline symptom severity, although regression to the mean cannot be excluded. Importantly, these observations do not diminish the clinical relevance of these factors: reducing loneliness, physical inactivity and somatic burden remain important aspects of depression management and are increasingly emphasised in clinical guidelines.^22 23^ Unlike symptom change, associations with diagnostic chronicity were attenuated by social and lifestyle factors, suggesting these domains may be more important for disorder persistence and represent potential targets for intervention.

The persistence of age-related differences in symptom trajectories further suggests that chronological age may capture mechanisms beyond those reflected in the measured covariates. Although indicators of somatic health such as the number of chronic diseases and BMI were included, these measures may not fully reflect aspects of physical burden most relevant to depressive chronicity, including disease severity, functional impairment or the psychological stress associated with managing long-term conditions.^24^ In addition, ageing-related biological processes, such as dysregulation of stress-response systems, inflammation-related dopamine depletion, vascular changes and reduced neuroplasticity, have been implicated in the pathophysiology of depression and may contribute to slower recovery at older ages.^25^

We further examined whether treatment-related factors could account for the observed age associations. Given that pharmacological treatment is more common at older ages whereas psychotherapy is more frequently received by younger individuals,^26^ differences in treatment exposure might have been expected to contribute to age-related differences in prognosis. Although associations remained after adjustment for treatment-related factors, it remains possible that older individuals experience reduced responsiveness to standard treatments, and that the potential of available strategies, including psychotherapy and other treatments (eg, dopamine-stimulating agents and transcranial magnetic stimulation), may not be fully realised in this patient group.^27–29^

This study has several strengths, including a large well-characterised clinical cohort with repeated assessments over 6 years, the combination of standardised diagnostic interviews, life chart data and psychopathology measures enabling nuanced classification of disease course, and extensive baseline data on clinical, somatic, lifestyle and social characteristics allowing systematic evaluation of potential explanatory factors. Several limitations should also be considered. Although follow-up rates were relatively high for a clinical cohort, some attrition occurred over time. Importantly, age distribution did not differ between participants retained throughout follow-up and those who dropped out (online supplemental Table S1), and there was no evidence that associations between prognostic factors and attrition differed by age (data not shown), reducing the concern about selective attrition by age. Second, the sample does not include individuals older than 65 years, precluding generalisation to older populations in whom age-related differences in prognosis may be even more pronounced,^11^ given even higher chronicity and lower remission rates of depression in late life.^13^ Third, although we adjusted for antidepressant use and psychotherapy at baseline, the absence of more detailed information on inadequate treatment response limits our ability to determine whether age-related differences in course partly reflect differential treatment responsiveness. Last, although metabolic syndrome indicators did not predict symptom course in the current sample,^30^ we did not additionally test other biological processes potentially underlying the observed age associations, which remain relevant objectives for future studies.

In conclusion, this 6-year longitudinal study showed that higher chronological age was an independent risk factor of a poorer course of depression, while age-related differences in anxiety prognosis appear more limited and confined to somatic symptom dimensions. These effects were not accounted for by somatic burden, social factors, lifestyle or treatment-related characteristics, suggesting that additional ageing-related biological or psychological processes may underlie these age differences.

### Clinical implications

These findings underscore the importance of sustained clinical attention to depression across the lifespan and highlight the need for future research to identify the specific mechanisms through which ageing confers prognostic risk. Clinically, the results call for a more proactive and potentially age-adapted approach to the management of depression in older adults, including closer follow-up, awareness of elevated risk of treatment resistance and consideration of novel therapeutic strategies.

## Data Availability

Data may be obtained from a third party and are not publicly available. The data used in this study are available from NESDA on reasonable request. Information on how to request access to the data, including the data sharing policy, is available at https://www.nesda.nl/nesda-english/ or by contacting the NESDA management team at nesda@amsterdamumc.nl.

## References

[1] Verduijn J, Verhoeven JE, Milaneschi Y, et al. Reconsidering the prognosis of major depressive disorder across diagnostic boundaries: full recovery is the exception rather than the rule. BMC Med 2017;15:215. 10.1186/s12916-017-0972-8 PMC5725897 29228943

[2] Herrman H, Patel V, Kieling C, et al. Time for united action on depression: a Lancet–World Psychiatric Association Commission. The Lancet 2022;399:957–1022. 10.1016/S0140-6736(21)02141-3 35180424

[3] Penninx BWJH, Nolen WA, Lamers F, et al. Two-year course of depressive and anxiety disorders: results from the Netherlands Study of Depression and Anxiety (NESDA). J Affect Disord 2011;133:76–85. 10.1016/j.jad.2011.03.027 21496929

[4] Bot M, Ligthart SA, Burger H, et al. Stable remission of depression after antidepressant treatment: prevalence and related sociodemographic and clinical characteristics in a longitudinal, real-world cohort. J Affect Disord 2026;398:121011. 10.1016/j.jad.2025.121011 41429327

[5] Buckman JEJ, Saunders R, Stott J, et al. Role of age, gender and marital status in prognosis for adults with depression: An individual patient data meta-analysis. Epidemiol Psychiatr Sci 2021;30:e42. 10.1017/S2045796021000342 PMC7610920 34085616

[6] Batelaan NM, de Graaf R, Spijker J, et al. The course of panic attacks in individuals with panic disorder and subthreshold panic disorder: a population-based study. J Affect Disord 2010;121:30–8. 10.1016/j.jad.2009.05.003 19477529

[7] Hardeveld F, Spijker J, De Graaf R, et al. Recurrence of major depressive disorder and its predictors in the general population: results from the Netherlands Mental Health Survey and Incidence Study (NEMESIS). Psychol Med 2013;43:39–48. 10.1017/S0033291712002395 23111147

[8] Spijker J, Bijl RV, de Graaf R, et al. Determinants of poor 1-year outcome of DSM-III-R major depression in the general population: results of the Netherlands Mental Health Survey and Incidence Study (NEMESIS). Acta Psychiatr Scand 2001;103:122–30. 10.1034/j.1600-0447.2001.103002122.x 11167315

[9] Ten Have M, de Graaf R, van Dorsselaer S, et al. Recurrence and chronicity of major depressive disorder and their risk indicators in a population cohort. Acta Psychiatr Scand 2018;137:503–15. 10.1111/acps.12874 29577236

[10] Ten Have M, Tuithof M, Velek P, et al. The Onset, Course and Co-Occurrence of Depressive and Anxiety Disorders Over a 9-Year Period in the General Population. Int J Methods Psychiatr Res 2026;35:e70054. 10.1002/mpr.70054 PMC12816878 41555769

[11] Schaakxs R, Comijs HC, Lamers F, et al. Associations between age and the course of major depressive disorder: a 2-year longitudinal cohort study. Lancet Psychiatry 2018;5:581–90. 10.1016/S2215-0366(18)30166-4 29887519

[12] Mitchell AJ, Subramaniam H. Prognosis of depression in old age compared to middle age: a systematic review of comparative studies. Am J Psychiatry 2005;162:1588–601. 10.1176/appi.ajp.162.9.1588 16135616

[13] Licht-Strunk E, van der Windt DAWM, van Marwijk HWJ, et al. The prognosis of depression in older patients in general practice and the community. A systematic review. Fam Pract 2007;24:168–80. 10.1093/fampra/cml071 17237495

[14] Batelaan NM, Rhebergen D, Spinhoven P, et al. Two-year course trajectories of anxiety disorders: do DSM classifications matter? J Clin Psychiatry 2014;75:985–93. 10.4088/JCP.13m08837 24912140

[15] Ramsawh HJ, Raffa SD, Edelen MO, et al. Anxiety in middle adulthood: effects of age and time on the 14-year course of panic disorder, social phobia and generalized anxiety disorder. Psychol Med 2009;39:615–24. 10.1017/S0033291708003954 PMC3679349 18667095

[16] Scholten W, Ten Have M, van Geel C, et al. Recurrence of anxiety disorders and its predictors in the general population. Psychol Med 2023;53:1334–42. 10.1017/S0033291721002877 PMC10009370 34294172

[17] Penninx BWJH, Beekman ATF, Smit JH, et al. The Netherlands Study of Depression and Anxiety (NESDA): rationale, objectives and methods. Int J Methods Psychiatr Res 2008;17:121–40. 10.1002/mpr.256 PMC6878352 18763692

[18] Solis EC, van Hemert AM, Carlier IVE, et al. The 9-year clinical course of depressive and anxiety disorders: New NESDA findings. J Affect Disord 2021;295:1269–79. 10.1016/j.jad.2021.08.108 34706441

[19] Schaakxs R, Comijs HC, Lamers F, et al. Age-related variability in the presentation of symptoms of major depressive disorder. Psychol Med 2017;47:543–52. 10.1017/S0033291716002579 27786143

[20] Hegeman JM, Kok RM, van der Mast RC, et al. Phenomenology of depression in older compared with younger adults: meta-analysis. Br J Psychiatry 2012;200:275–81. 10.1192/bjp.bp.111.095950 22474233

[21] Bekhuis E, Boschloo L, Rosmalen JGM, et al. Differential associations of specific depressive and anxiety disorders with somatic symptoms. J Psychosom Res 2015;78:116–22. 10.1016/j.jpsychores.2014.11.007 25524436

[22] National Institute for Health and Care Excellence. Depression in adults with a chronic physical health problem: recognition and management. 2009. 39808015

[23] National Institute for Health and Care Excellence. Depression in adults: treatment and management. 2022. 35977056

[24] Gold SM, Köhler-Forsberg O, Moss-Morris R, et al. Comorbid depression in medical diseases. Nat Rev Dis Primers 2020;6:69. 10.1038/s41572-020-0200-2 32820163

[25] Marx W, Penninx BWJH, Solmi M, et al. Major depressive disorder. Nat Rev Dis Primers 2023;9:44. 10.1038/s41572-023-00454-1 37620370

[26] Jokela M, Batty GD, Kivimäki M. Ageing and the prevalence and treatment of mental health problems. Psychol Med 2013;43:2037–45. 10.1017/S0033291712003042 23324208

[27] Valiengo L, Maia A, Cotovio G, et al. Repetitive Transcranial Magnetic Stimulation for Major Depressive Disorder in Older Adults: Systematic Review and Meta-analysis. J Gerontol A Biol Sci Med Sci 2022;77:851–60. 10.1093/gerona/glab235 34432865

[28] Cuijpers P, Karyotaki E, Eckshtain D, et al. Psychotherapy for Depression Across Different Age Groups: A Systematic Review and Meta-analysis. JAMA Psychiatry 2020;77:694–702. 10.1001/jamapsychiatry.2020.0164 PMC7081149 32186668

[29] Jeuring HW, D’Angremont E, Tol MH, et al. The effectiveness of off-label dopamine stimulating agents in depressive disorder: A systematic review and meta-analysis. Psychiatry Res 2023;319:115010. 10.1016/j.psychres.2022.115010 36528007

[30] Hiles SA, Révész D, Lamers F, et al. Bidirectional prospective associations of metabolic syndrome components with depression, anxiety and antidepressant use. Depress Anxiety 2016;33:754–64. 10.1002/da.22512 PMC5111740 27120696

